# Current Status and Future Perspectives of Preoperative and Intraoperative Marking in Thoracic Surgery

**DOI:** 10.3390/cancers16193284

**Published:** 2024-09-26

**Authors:** Toyofumi Fengshi Chen-Yoshikawa, Shota Nakamura, Harushi Ueno, Yuka Kadomatsu, Taketo Kato, Tetsuya Mizuno

**Affiliations:** Department of Thoracic Surgery, Nagoya University Graduate School of Medicine, Nagoya 466-8550, Japan; shota197065@med.nagoya-u.ac.jp (S.N.); h-ueno@med.nagoya-u.ac.jp (H.U.); ykadomatsu@med.nagoya-u.ac.jp (Y.K.); tkato@med.nagoya-u.ac.jp (T.K.); te.mizuno@med.nagoya-u.ac.jp (T.M.)

**Keywords:** intraoperative marking, marker-less marking, molecular imaging, preoperative marking, surgical simulation, three-dimensional imaging, thoracic surgery, video-assisted thoracoscopic surgery

## Abstract

**Simple Summary:**

With advances in radiological imaging and its increased use, small nodules are being detected more frequently. Surgical resection is often the final option for diagnosis and treatment; however, small nodules may be too small to be detected during surgery. The lungs are soft, deformable organs that can change shape during respiratory phases and surgical procedures. Thus, surgeons rely on preoperative or intraoperative markings for nodule identification and precise resection. Furthermore, two randomized clinical trials found that sublobar resection could be an alternative treatment for early-stage non-small-cell lung cancer. Therefore, the demand for preoperative or intraoperative marking techniques is increasing, particularly for wedge resection or segmentectomy. In this study, we provide a narrative review of the current status and future perspectives of preoperative and intraoperative markings in thoracic surgery.

**Abstract:**

The widespread implementation of lung cancer screening and thin-slice computed tomography (CT) has led to the more frequent detection of small nodules, which are commonly referred to thoracic surgeons. Surgical resection is the final diagnostic and treatment option for such nodules; however, surgeons must perform preoperative or intraoperative markings for the identification of such nodules and their precise resection. Historically, hook-wire marking has been performed more frequently worldwide; however, lethal complications, such as air embolism, have been reported. Therefore, several surgeons have recently attempted to develop novel preoperative and intraoperative markers. For example, transbronchial markings, such as virtual-assisted lung mapping and intraoperative markings using cone-beam computed tomography, have been developed. This review explores various marking methods that have been practically applied for a better understanding of preoperative and intraoperative markings in thoracic surgery. Recently, several attempts have been made to perform intraoperative molecular imaging and dynamic virtual three-dimensional computed tomography for the localization, diagnosis, and margin assessment of small nodules. In this narrative review, the current status and future perspectives of preoperative and intraoperative markings in thoracic surgery are examined for a better understanding of these techniques.

## 1. Introduction

Lung cancer is one of the most common and lethal neoplasms and the leading cause of cancer-related deaths [1]. Thoracic surgery remains the most effective therapeutic option despite advancements in noninvasive treatments. Reflecting on the history of surgical treatment of lung cancer, the first successful left pneumonectomy for lung cancer was performed by Graham and Singer in 1933 [2]. Approximately 30 years later, Cahan et al. first proposed radical lobectomy for the surgical treatment of lung cancer in 1965 [3]. Approximately 30 years later, in 1995, Ginsberg and Rubinstein reported a randomized Lung Cancer Study Group 821 trial, which compared lobectomy to limited resection for early-stage lung cancer, finding unfavorable results for sublobar resection [4]. Since then, lobectomy has become the standard of care for all patients with isolated primary lung cancer. However, many thoracic surgeons have pursued sublobar resection as a treatment option for small non-small cell lung cancer (NSCLC). The rate of sublobar resection, including wedge resection and segmentectomy, for lung cancer in Japan increased from 6.4% in 1994 to 22.7% in 2010 [5]. More recent Japanese national registry data revealed that the rate of sublobar resection became 32.9% (8683 wedge resections and 6781 segmental resections) of all lung cancer surgeries (46,624 surgical resections) in 2021 [6].

According to the validation of the efficacy of lung cancer screening and the increasing use of thin-slice computed tomography (CT) worldwide, an increasing number of small nodules, including ground-glass nodules, can be frequently detected; therefore, these nodules have more often been addressed by respirologists or thoracic surgeons [7,8]. There is a possibility that these nodules are lung cancer, which is the most lethal malignant lesion worldwide; therefore, thoracic surgeons are forced to perform a resection, based on the patient’s desire, to make a diagnosis and perform a curative resection using a surgical procedure [9]. However, there is a high possibility that such nodules are so small that they cannot be detected during surgery. The lungs are soft and deformable organs owing to the respiratory phases and surgical procedures; therefore, what is seen during surgery differs from preoperative CT [10]. Therefore, it is important for surgeons to employ preoperative or intraoperative markings for the identification of nodules and their precise resection [11].

Two randomized trials have found that sublobar resection can be an alternative or even superior treatment for early-stage small NSCLC compared to conventional lobectomy [12,13]. In this regard, the use of wedge resection or segmentectomy during surgery is expected to increase, instead of lobectomy, which requires more preoperative or intraoperative markings because it is difficult to identify such small nodules as they are sometimes invisible and unpalpable [8,9]. Furthermore, there has been a steady transition toward minimally invasive surgical approaches to the treatment of lung cancer, including video-assisted thoracic surgery (VATS) and robot-assisted thoracic surgery (RATS). In minimally invasive approaches, small nodules with ground-glass opacification (GGO) or those located deep within the lung parenchyma are more likely to present technical challenges in the intraoperative localization and/or assessment of resection margins [14,15].

To date, various techniques for localizing pulmonary nodules have been developed for their diagnosis and treatment all over the world [16]. Historically, hook-wire marking, which is a well-known percutaneous marking, has been more frequently performed worldwide; however, lethal complications such as air embolization have been reported in several institutions [17,18]. This type of lethal complication rarely occurs, but its outcome is disastrous. Therefore, several surgeons have recently attempted to develop novel preoperative and intraoperative markers. For example, transbronchial and intraoperative markings using cone-beam computed tomography (CBCT) have been performed more often [8,10,19,20]. However, these types of markings have not been as widely used as hook-wire markings. Therefore, we conducted a narrative review to understand the current status and future perspectives of preoperative and intraoperative marking in thoracic surgery.

## 2. Preoperative Marking

### 2.1. Clinical Overview and Three-Dimensional Reconstruction

The need for sublobar resection has increased rapidly worldwide owing to the development of surgical and radiological techniques. Furthermore, smaller nodules are more likely to be resected using a less invasive method. As a result, thoracic surgeons are more frequently required to perform preoperative markings to ensure precise surgical resection, especially when performing sublobar resection [21]. Preoperative marking started with marking only small nodules, but now includes marking the corresponding intersegmental plane as well as the targeted nodule. Therefore, three-dimensional (3D) reconstruction technology and its recent advancements should be emphasized. Thoracic surgeons have primarily relied on two-dimensional (2D) computed tomography (CT) images to plan the identification of nodules and their precise resections with appropriate margins. After the comparison of 2D CT and 3D CT imaging, Bakhuis and colleagues found that the actual nodule site differed from what had been predicted using 2D CT images in 14% of cases [22]. Interestingly enough, comparative studies consistently demonstrated that a lower incidence of inadequate margins when preoperative planning incorporated 3D CT images, in comparison with relying only on 2D CT images [22,23]. Furthermore, Bakhuis and colleagues also found that the treatment plan required adjustments in 52% of the patients to ensure adequate margins [22]. Recent advancements in 3D imaging reconstruction technology enable thoracic surgeons to construct accurate 3D images of patients from their preoperative CT without difficulties [10]. These images can be widely used at the time of preoperative and intraoperative markings [8,9].

### 2.2. Percutaneous Marking

In preoperative percutaneous marking, needle localization techniques are generally used, in which a guidewire is placed percutaneously under CT guidance. Several types of guidewires, such as hook wires, microcoils, and spiral wires, are globally available [21]. Historically, hook-wires have been widely used. Instead of hook wires, various types of dyes or substances, such as lipiodol, silicone, and collagen, have been used as alternative markers [24,25,26]. More recently, Doncic and colleagues introduced a technique of radiotracer with sufficient results in 57 patients, in which gamma-probe detection was performed during surgery after preoperative percutaneous marking of the nodule using technetium-99m labelled albumin macro-aggregates [27]. They reported that precise detection and resection was possible in 95% of the lesions and in 93% of the patients with minor complications in 23% of the patients who did not require additional interventions. Voulaz and colleagues also introduced preoperative percutaneous CT-guided localization with indocyanine green (ICG) for the thoracoscopic resection of small pulmonary nodules in 40 patients with sufficient outcomes [28]. They reported that all pulmonary nodules were easily detected and successfully resected without any complications throughout the whole procedure.

**Figure 1:**
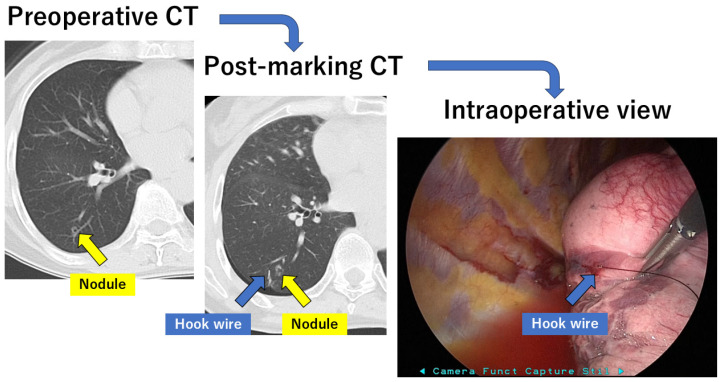
Preoperative marking with hook wire. CT: computed tomography.

In the hook-wire method, marking is performed a few hours before the surgery on the same day, under the guidance of chest CT (Figure 1). Surgeons can easily identify localized sites intraoperatively without additional radiation exposure. This method has been established worldwide and most radiologists and thoracic surgeons can perform the procedure. The success rate of CT-guided hook-wire marking has been reported to be as high as 92.5–97.6%, although some were either dislodged or displaced [29,30,31,32]. The dislodging rate of the hook wire is reported to be 2.4–7.5%. Another disadvantage is the risk of complications. Pneumothorax was the most common complication. A slight pneumothorax is the most common complication, occurring in 7.5–40% of cases [29,30,31,32]. Lung hemorrhage occurs at a rate of 10.3–36% [29,30,31,32]. Air embolism is the most lethal complication [18,33]. This complication is extremely rare; however, if it occurs, it will prevent the physician in charge from performing this preoperative marking.

Furthermore, percutaneous marking could not be performed owing to the anatomical location of the lesions. For example, it would be difficult to place a precise preoperative marking on the tumor beneath the scapula or on the mediastinal side.

### 2.3. Transbronchial Marking

To resolve the potential disadvantages, including the complications of percutaneous marking, transbronchial marking has been developed. Various types of markers were delivered endoscopically, such as dye agents, coils, and fiducials [34]. The choice of dye agent depends on several factors, but one of the major factors is the time between the procedure for localization and surgery. It is reported that indigo carmine lasts a few days [20,35,36,37], while methylene blue can diffuse to adjacent tissues relatively more rapidly [38,39].

**Figure 2:**
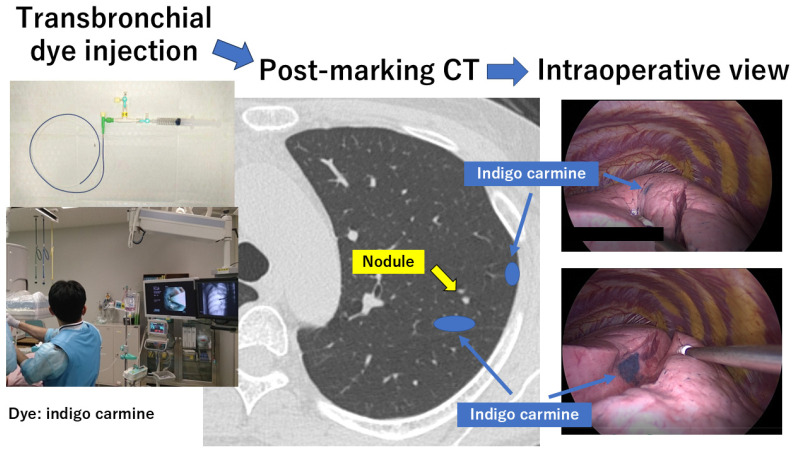
Virtual-assisted lung mapping (VAL-MAP). CT: computed tomography.

Sato et al. invented virtual-assisted lung mapping (VAL-MAP) at Kyoto University in 2012 [20,35,36,37]. The VAL-MAP consists of planning, dye injection under bronchoscopy, CT after mapping, and surgery (Figure 2). First, 3D-CT images and virtual bronchoscopy images were obtained from the patient’s CT data. Lung mapping was planned to identify a nodule and indicate the resection line for sublobar resection. Multiple markings are typically considered, although the number of markings depends on the purpose and characteristics of the surgery. Second, the bronchoscopic procedure was performed under local anesthesia and mild sedation on the day of surgery or one day before surgery. Once the bronchoscope reached the target bronchus using the predefined virtual bronchoscopy route, a small metal-tip catheter preloaded with indigo carmine (Daiichi-Sankyo, Tokyo, Japan) was inserted into the bronchus and advanced to the visceral pleura under the guidance of fluoroscopy. When the tip of the catheter reached the visceral pleura through the target bronchus, approximately 4 mg (1 mL) of indigo carmine was injected into the target bronchus through the small metal-tip catheter. This procedure was repeated to complete all the planned markings for multiple mapping. This bronchoscopic marking procedure is usually performed within a few days before surgery. Third, post-mapping CT was taken and 3D-CT images were reconstructed to confirm the actual locations of the nodules and corresponding marks. These CT scans for 3D CT images are usually taken within a few hours after the mapping procedures. During surgery, the dye marks can be recognized as blue spots on the surface of the lung, and surgical resection can be performed easily. With regard to adverse events, VAL-MAP has lower complication rates than the percutaneous methods. In fact, minor pneumothoraces without symptoms were found on post-mapped CT images in only 4 out of 100 patients, which did not require any additional treatment [35]. Compared to the percutaneous marking method, VAL-MAP can be used to perform multiple markings for multiple lesions without adverse effects, including pneumothoraces. Multiple markings can provide geometric information on the lungs, enabling the application of VAL-MAP for segmentectomy. VAL-MAP can help identify intersegmental planes and resection margins more accurately; therefore, it can be used more frequently for extended or complex segmentectomies [40]. VAL-MAP was widely performed in Japan and is currently being performed in some Asian countries. However, precise transbronchial marking is sometimes difficult, which may become a hurdle for physicians who are unfamiliar with transbronchial interventions. Even for a professional physician familiar with bronchoscopy, there is a possibility that markings cannot be easily identified on post-marking CT and/or at the time of surgery. According to the initial experience reported in 2015 [35], 22.6% of the markings were difficult to identify on post-marking CT and 16.2% of the markings were difficult to identify during surgery.

**Figure 3:**
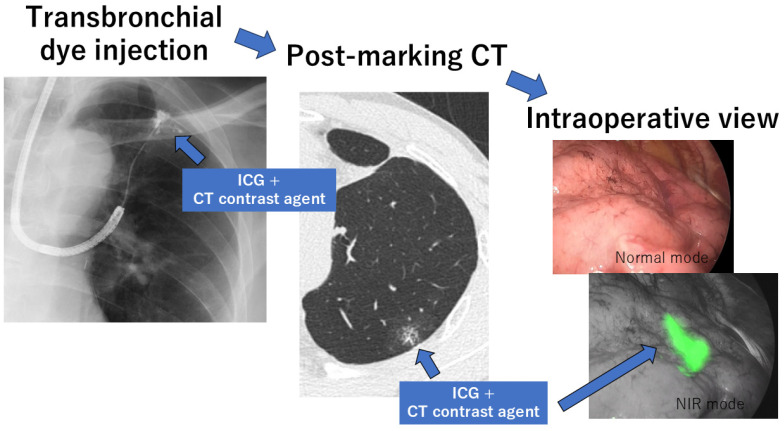
Indocyanine green virtual-assisted lung mapping (ICG-VAL-MAP). CT: computed tomography, NIR: near infrared.

To overcome these disadvantages of VAL-MAP, ICG was used instead of indigo carmine [41,42] (Figure 3). Tokuno and Chen-Yoshikawa used a mixture of ICG and a CT contrast agent (Iopamiron; Bayer AG, Leverkusen, Germany) to improve the visibility of the markings [41]. In this method, called ICG-VAL-MAP, a CT contrast agent is used to improve the visibility of the markings on post-marking CT. Conversely, ICG is used to improve the visibility of the markings during surgery. They demonstrated that the rate of easily identifiable markings on post-marking CT images increased from 77% to 100% with the introduction of ICG-VAL-MAP. Furthermore, the rate of easily identifiable markings during surgery increased from 83.8% to 99.3% with the introduction of the ICG-VAL-MAP. More recently, Nagano and Sato reported that the successful resection rate of small lung nodules was >97% when three or more VAL-MAP marks were identified during surgery [43]. In VAL-MAP, there is a possibility that markings cannot be identified, but an increase in the number of VAL-MAP marks may cause complications. Yanagiya et al. performed VAL-MAP dual-staining using both ICG and indigo carmine with satisfactory results [42,44]. Although VAL-MAP using ICG may be considered more useful, this method requires a near-infrared scope. Furthermore, ICG cannot be used in patients with iodine hypersensitivity.

Another disadvantage of VAL-MAP is the difficulty of acquiring an appropriate resection margin for deeply located nodules. To overcome this issue, VAL-MAP 2.0 was developed, in which a microcoil is placed transbronchoscopically in addition to dye marking. A multicenter prospective study revealed that the successful resection rate was as high as 98.5%, although 3 out of 75 (4%) microcoils showed major displacement after bronchoscopic placement [45]. However, in this procedure, a fluoroscope must be used several times during surgery and microcoils are expensive. Simultaneously, a novel wireless localization technique using radiofrequency identification (RFID) marking has been developed, which does not require palpation of the lung [46,47,48]. A multicenter study in Japan proved that the RFID lung marking system was safe and effective during successful sublobar resection and that patients with pure ground-glass nodules would be the best candidates for this system [48]. Additionally, RFID markers provide accurate positional information with a depth similar to VAL-MAP 2.0 [46].

In these procedures, the bronchoscopic technique is important; therefore, electromagnetic bronchoscopy (ENB) has also been used for precise marking [46,49]. A combination of VAL-AMP and ENB has been reported, in which onsite adjustment with ENB can eliminate the post-mapping CT scan requirement [49]. In addition, ENB-guided RFID marker placement ensured adequately deep surgical margins [38]. Robot-assisted bronchoscopy can be used to identify lung nodules accurately [50]. However, although precise marking can be performed, these methods require expensive modalities. More recently, lung nodules marked with ICG dye-soaked coils have been reported [51]. In this method, all patients underwent robot-assisted navigation bronchoscopy with either CBCT or three-dimensional fluoroscopy as an additional tool for confirmation.

## 3. Intraoperative Marking

Intraoperative marking is performed in the operating room immediately before surgery and has been widely adopted. Theoretically, both percutaneous and transbronchial markings written in the preoperative marking section can be performed intraoperatively. The advantages of this method include a simplified workflow with immediate transition to surgical resection in the same operating room, high accuracy, complete control of ventilation, and no pain associated with the marking procedure.

**Figure 4:**
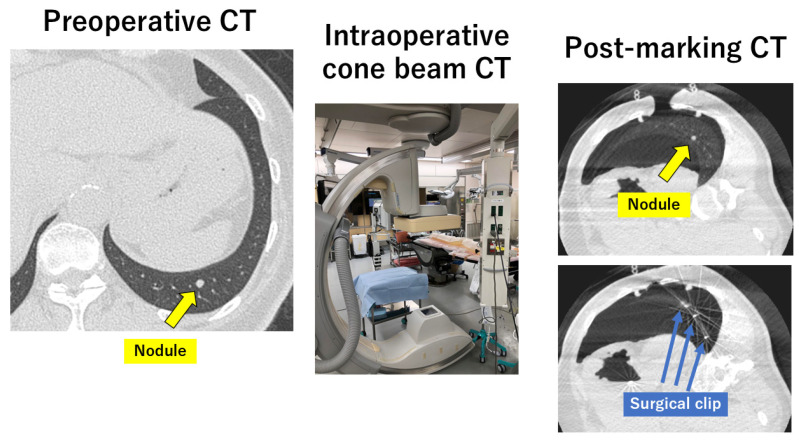
Intraoperative marking with cone beam computed tomography (CBCT). NIR: near-infrared.

Another new technique is the use of cone beam CT (CBCT) in a hybrid operating room, which is called the CBCT method [19,52] (Figure 4). For example, after the induction of general anesthesia, patients are placed in the lateral decubitus position. The CBCT C-arm and the patient’s chest are protected with sterile wraps, and an initial scan for surgical planning is acquired using CBCT. After outlining the needle path by marking the needle entry and target points, puncture of the lung parenchyma, followed by dye injection, is performed near the nodule. The usual VATS procedure is then conducted for the precise surgical resection of the nodule under the guidance of the injected dye marking [53]. Gilberto and colleagues reported a successful lung nodule localization in a hybrid room before minimally invasive thoracic surgery in 20 patients using a variety of markers, such as guidewire, lipiodol, and microcoil [54]. They reported that all lesions were identified and resected completely despite two cases of pneumothorax, one case of coil displacement, and one case of hypotension after marking. Lyberis and colleagues also demonstrated all-in-one diagnostic and therapeutic precision thoracic surgery in a hybrid operating room using triple marking technique with gold seeds, methylene blue, and ICG [55]. They reported that they were able to correctly identify the lung lesions in all the patients by visual identification thanks to the use of at least one marking technique.

Instead of dye marking before the VATS approach, another method can be performed using surgical clips attached to the visceral pleura around the suspected location of the nodule based on the preoperative CT image using a three-port VATS approach [56,57]. In a strict sense, this is also true for intraoperative markings. Moreover, it has been reported that intraoperative marking for the identification of intersegmental planes in pulmonary segmentectomy can be performed with high accuracy and convenience using a combination of the intraoperative injection of ICG and CBCT [52].

Additionally, ENB can be used with or without CBCT for preoperative markings [58]. In comparison with conventional CT-guided marking, the distinctive features of ENB-guided marking are that ENB-guided procedures can be easily performed in the operating room under general anesthesia just before thoracoscopic surgery [58,59]. ENB can guide accurate cryobiopsy and dye marking before lung resection [60]. Intraoperative pathological diagnosis can be obtained through ENB-guided transbronchial lung cryobiopsy, which avoids the need for diagnostic wedge resection. Simultaneous ENB-guided dye marking can facilitate precise lung resection. Furthermore, robotic-assisted bronchoscopy with CBCT ICG dye marking for lung nodule localization has been reported to have higher accuracy and superior maneuverability than that of ENB procedures [61].

Moreover, intraoperative ultrasound can be a useful tool for the detection of small lungs and ground-glass nodules [62]. Intraoperative ultrasonography is useful as an alternative to manual palpation for determining the accurate location of pulmonary nodules during RATS [63].

## 4. Molecular Imaging

Intraoperative molecular imaging is a novel technique aimed at the localization, diagnosis, and margin assessment of lung nodules. Although several promising methods have been developed, they have not yet been widely used [21]. ICG was the first commercially available agent by the Food and Drug Administration, which was non-targeted and thought to localize tumors through increased vasculature and dysfunctional lymphatics. Okusanya reported that ICG localized 16 out of 18 preoperatively diagnosed small pulmonary nodules, with sizes as small as 0.2 cm and depths up to 1.3 cm from the pleural surface [64]. Another group also reported that 95% (37 out of 39) of non-small cell lung cancer with a consolidation-to-tumor (C/T) ratio of >50% could be successfully detected by ICG; however, ICG failed in 12 non-small cell lung cancers with a C/T ratio of <50%, which means that further research is required to develop fluorescent agents targeting lung cancer, especially for ground-glass nodules [65]. In contrast, Hamaji reported that ICG could identify some metastatic tumors, but not all such tumors [66]. Thereafter, folate-targeted dyes, such as EC 17 and OTL 38, localizing to pulmonary adenocarcinomas that highly express folate receptor alpha, have been developed [67,68]. OTL-38 is currently considered as one of the most promising technologies for real-time subcentimeter pulmonary localization, margin assessment, and intraoperative diagnosis [69,70]. Kennedy and his colleagues documented their single-institution experience with the first 500 pulmonary resections guided by intraoperative molecular imaging [71].

## 5. Real-World Data in a Single Institution

The authors herein present clinical data from Nagoya University Hospital, where they are affiliated, as real-world data. The status of preoperative and intraoperative markings for four years, from 2020 to 2023, at Nagoya University Hospital is detailed in Table 1. In our hospital, approximately 450 surgeries are performed annually under general anesthesia and 300–400 of them are performed for lung malignancies. Until 2019, the hook-wire method was the only preoperative marking method. In October 2020, the VAL-MAP and CBCT methods were introduced. Since then, three types of markings have become available.

Indications for preoperative and intraoperative markings vary from case to case as well as from time to time, but decisions are finally made by the thoracic surgeon in charge after repetitive discussions at preoperative conferences held by thoracic surgeons. In general, the VAL-MAP method can be performed for almost all nodules, but the hook-wire method is neither chosen for nodules in the lungs behind the scapula nor those on the mediastinal or diaphragmatic side. However, when a nodule is located away from the pleura, it is difficult to identify the nodule and/or to perform sublobar resection with enough margin, even when using preoperative and intraoperative markings. In such cases, lobar resection cannot be used for the exact diagnosis and precise surgical treatment. In multiple nodules or emphysematous lungs, there is a high possibility that the hook-wire method would be avoided because of the possible pneumothorax during and/or after the marking.

Table 2 describes the conditions of each marking method in our hospital. The hook-wire method was performed by a radiologist one day before surgery or on the day of surgery. Thoracic surgeons are required to be present during the marking procedure, which can be performed almost daily. The VAL-MAP method, performed by a respirologist one day before surgery, requires thoracic surgeons to be present in the bronchoscopy suite. The VAL-MAP can be performed only one day per week. The CBCT can be performed by thoracic surgeons on the day of surgery; however, this method requires a hybrid operating room where an intraoperative CT scan can be performed. A special hybrid operating room should be reserved in advance. Furthermore, a radiologic technician familiar with special fluoroscopy in the hybrid operating room is also necessary for manipulating CBCT. In addition, hybrid operating rooms are regularly used by cardiac surgery, vascular surgery, and neurosurgery teams. Thoracic surgery can make reservations for this room only one day per week, but other teams have greater priority for reservations, which means that this room is often unavailable to thoracic surgeons. The CBCT should be used in several special settings during surgery, including rotating a C-arm imaging device in a clean field, which means that thoracic surgeons are often unfamiliar with this method, unwilling, or reluctant to choose it for marking. Thoracic surgeons found the hook-wire method the easiest to perform, followed by the VAL-MAP method. The CBCT method is more challenging than the other two methods.

During the four-year study period, markings were performed in 124 patients. Of the 124 patients, preoperative and intraoperative markings were performed in 119 patients for pulmonary malignancies and in only 5 patients for pulmonary benign diseases, such as inflammatory granuloma, intrapulmonary lymph node, pulmonary cryptococcosis, alveolar cell hyperplasia, and follicular bronchitis (Table 1). The patients who underwent preoperative or intraoperative marking comprised approximately 10% of the annual number of surgeries performed for lung malignancies. Since the VAL-MAP and CBCT methods were first used in 2020, there were relatively more patients with markings by VAL-MAP and CBCT in 2020 and 2021, respectively; however, the number of CBCT methods decreased dramatically by 2023. Currently, the hook wire and VAL-MAP methods are the main options for marking. The hook-wire method is performed percutaneously; therefore, marking a nodule in the lungs behind the scapula is not routinely possible, nor is marking a nodule on the mediastinal side possible. For such nodules, the VAL-MAP method can be appropriately used. Furthermore, a combination of the two markings was performed simultaneously in 14 patients, and additional marking was useful in most patients. In summary, this trend in the three markings reflects the availability of these methods at our hospital.

## 6. Future Perspectives

As discussed in this manuscript, various marking methods have been developed to can accurately identify smaller nodules and perform precise surgery; however, CT-guided hook-wire localization, which is one of the oldest techniques, remains the most frequently used technique despite several drawbacks, including lethal complications. One of the main reasons many physicians use a hook wire for marking is its simplicity. Various types of markings have been developed with high accuracy; however, these techniques are complicated or require technical proficiency. The most popular technique is chosen based on physicians’ preferences in hospitals worldwide. One of the most important aspects for users of these techniques is how easy they are to use by themselves. Our institution provides a good example. In other words, we have not truly created an alternative marking method to hook wire at present.

**Figure 5:**
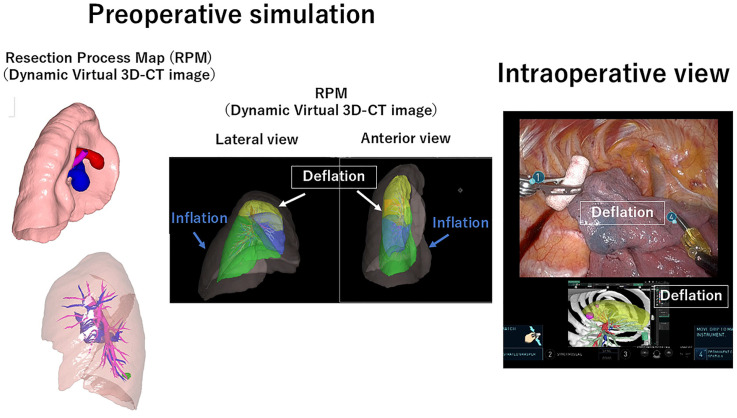
Resection process map (RPM). 3D-CT: three-dimensional computed tomography.

To overcome this issue, novel marking methods are required. It would be best if we could completely perform cancer-specific marking. However, another realistic option would be markerless marking using a 3D CT image with deformation by deflation and/or surgical manipulation. The technique of 3D CT reconstruction has been developed and is widely distributed worldwide and numerous commercialized software has also been utilized globally [10,72,73]. Furthermore, it has been reported that 3D CT images can be reconstructed even from nonenhanced CT data [10]. However, the current 3D CT images are static and do not correspond to deflation and/or surgical deformations. To incorporate deflated and surgical deformations of the lungs, an algorithm called the resection process map (RPM) (Figure 5) was developed [74]. This algorithm has been clinically evaluated, and the current 3D CT images can be converted into dynamic virtual 3D CT images for surgical navigation in the future [75,76,77]. It is expected that a small lung nodule could be identified without placing any preoperative or intraoperative markers using this novel algorithm, leading to the development of markerless marking, which could provide a novel alternative to current marking systems.

## 7. Conclusions

Surgeons are required to perform preoperative or intraoperative marking to accurately identify small suspicious nodules and perform a precise resection. Recently, various marking methods have been developed, which have been widely applied. They have been reviewed to provide a comprehensive understanding of the current status and future perspective of preoperative and intraoperative markings in thoracic surgery. More recently, several attempts have been made to perform intraoperative molecular imaging and dynamic virtual 3D CT imaging. These advancements aim to enhance the localization, diagnosis, and margin assessment of such nodules.

## Figures and Tables

**Table 1:** Trend of preoperative and intraoperative markings in Nagoya University Hospital. CBCT: cone beam computed tomography, CT: computed tomography, OR: operating room, VAL-MAP: virtual-assisted lung mapping.

Marking Method	Before 2019	2020	2021	2022	2023	Sum
Hook wire	All	15	8	18	21	62
VAL-MAP		2	11	12	12	37
CBCT		5	20	13	1	39
(Multiple: VAL-MAP + CBCT)		(2)	(4)	(7)	(1)	(14)
Patients undergoing preoperativeor intraoperative marking		20	35	36	33	124
Surgery for malignant tumors		305	325	342	370	1342
(Rate of marking in surgery for malignant tumors)		(7%)	(11%)	(11%)	(9%)	(9%)

**Table 2:** Comparison between methods for the preparation of each procedure. CBCT: cone beam computed tomography, CT: computed tomography, OR: operating room, VAL-MAP: virtual-assisted lung mapping.

Marking Method	Place	Performer	Condition	Timing
Hook wire	CT suite	Radiologist	With thoracic surgeon	Almost every day (One day before or on the day of surgery)
VAL-MAP	Bronchoscopy suite	Respirologist	With thoracic surgeon	Once a week (Thursday) (One day before surgery)
CBCT	Hybrid OR	Thoracic surgeon	When not used by other departments with priority rights	Once a week (Friday) (On the day of surgery)
